# Alignment Between Classroom Education and Clinical Practice of Root Canal Treatment Among Dental Practitioners in China: Cross-Sectional Study

**DOI:** 10.2196/65534

**Published:** 2025-07-29

**Authors:** XinYue Ma, JingShi Huang

**Affiliations:** 1 Humanomics Science Center, International Institute of Creative Design, Shanghai University of Engineering Science, 350 Xianxia Road, Shanghai, 200335, China, 86 18402155120

**Keywords:** root canal treatment, endodontic education, dental education, clinical practice, dental medical workers

## Abstract

**Background:**

This cross-sectional study assessed the perceived alignment between preclinical education and clinical practice in root canal treatment (RCT) among dental practitioners in China, aiming to identify systemic gaps in dental curricula and their clinical implications.

**Objective:**

Dental professionals in Eastern Coastal China. This study distributed questionnaires through hospital dental specialties and medical forums, covering the Southeastern Region of China.

**Methods:**

A validated, web-based survey was distributed to 90 dental professionals in Eastern Coastal China, focusing on 9 key stages of RCT, preoperative preparation, intraoperative procedures, postoperative care, and clinician-patient communication. Responses were measured using a 7-point Likert scale to evaluate perceived discrepancies between education and clinical practice.

**Results:**

A total of 83 valid questionnaires were recovered, which revealed significant disparities between academic training and clinical demands. The survey showed that the specialized practitioners identified pronounced mismatches in RCT operative techniques and doctor-patient communication (*P*<.05). Participants aged ≤29 years demonstrated heightened awareness of discrepancies in disinfection protocols and temporary filling procedures (*P*<.05). Shanghai-trained practitioners reported fewer educational-clinical gaps across multiple procedural stages (*P*<.05). Notably, 82% of respondents rated comprehensive RCT implementation as more challenging than individual procedural components. Curriculum deficiencies were identified in treatment indication diagnostics (56.6% agreement) and communication training (43.4% agreement). Emerging technologies like virtual reality and augmented reality (VR and AR) showed minimal educational penetration (3.7% exposure rate). In the free-response section, qualitative feedback highlighted equipment accessibility issues (eg, thermal gutta-percha tools) and instructor-dependent learning outcomes.

**Conclusions:**

Structural discrepancies exist in Chinese preclinical RCT education, influenced by factors such as experience level, age, and region. These findings underscore the need for curriculum reforms, emphasizing competency-based training, enhanced simulation technologies, and standardized clinical protocols, particularly in areas like periodontal pathology and communication skills.

## Introduction

Periapical lesions and endodontic diseases are highly prevalent among the global adult population [1], with root canal treatment (RCT) being one of the most common interventions provided by general dentists in clinical practice and the preferred treatment method for treating endodontic diseases.

Studies have shown that RCT is a challenging clinical intervention in dentistry, leading to varying degrees of practical difficulty for dental practitioners. Over 70% of dental practitioners express a desire for more clinical training [2]. Unlike most surgical dental procedures, RCT involves multiple steps, and any errors can result in unsatisfactory outcomes. RCT is performed in a concealed space, with limited space for operation and without visual control. It involves a variety of equipment, requires fine competence of the dentist, and involves complex operational procedures. According to dental practitioners, they often perform RCT procedures feeling a lack of control. In an emotional survey report related to RCT, nearly all dentists expressed feelings such as anxiety, frustration, stress, or exhaustion [3].

Compared with other dental disciplines, endodontic therapy demands higher levels of manual dexterity and independent operational skills. Educational institutions play a crucial role in nurturing individuals to meet societal needs [4]. Optimal dental education should produce competent general practitioners in dentistry to ensure patient safety [5,6]. However, the difference in education among dental schools is considered a major obstacle to standardizing and ensuring the quality of dental education [5]. Early research on endodontic education focused on improving teaching methods, while later studies began to focus on different learning periods and the introduction of new technologies [7]. Authoritative organizations such as the European Society of Endodontology (ESE) have established guidelines to ensure educational standards. Furthermore, most dental schools in China offer a 5-year dental training program leading to a Bachelor of Dental Surgery degree, with the emphasis on clinical practice training in the final year of the dental education plan (following the “Clinical Practice for Chinese Undergraduate Students Majoring in Stomatology” standards established by Chinese Stomatological Association) [8]. Due to differences in economic and cultural backgrounds between Western countries and China, disparities exist in dental education programs, licenses, curricula, and facilities [9].

Regarding the allocation of teaching time in endodontic education, significant differences exist among countries. Previous studies show substantial variations in preclinical endodontic education at dental schools. In Germany, the average time spent on theoretical courses is 13.3 hours, practical courses require an average of 45.4 hours, and the total time for the endodontics course averages 56 hours [10,11]. In Spain, 95% of schools allocate over 20 hours for preclinical training, with 60% of schools dedicating over 50 hours [12]. However, in a study in China, 71.99% of Chinese schools spend less than 4 hours per week on endodontics education, totaling no more than 80 hours per semester, with the lowest training time allocated to periodontics and significant shortages in facilities for dental surgery courses [13]. This indicates that education emphasis and methods vary greatly across regions.

In terms of educational systems, comparing China and the United States illustrates 2 distinct teaching models. In the United States, after completing 4 years of general education, students are required to independently prepare some practice equipment, have relatively ample time to focus on coursework and preclinical training at schools, and enhance their skills through dental practice [9]. In China, dental schools are divided into three categories: 8-year program, 5-year program, and 3-year program. The 8-year program is only held in a few renowned dental schools in China, while the majority of dental students pursue the 5-year Bachelor program [14]. Although the order and proportion of courses may differ across schools in China, the undergraduate curriculum generally includes basic courses and dental courses. The undergraduates take public courses and basic courses of medicine in the first 2 years, focusing on clinical medical courses in the junior and senior years, with the opportunity to do a rotation in several departments such as Otolaryngology and Endocrinology. In the final year, there is no theoretical research, and all the time belongs to clinical practice. In America, clinical practice typically takes 2 years. In addition, China has 93 dental institutions offering a shorter 3-year training program for dental assistants [15]. Upon completion of the 3-year program and assessments, students are awarded the junior college in China, comparable to Bachelor of Science in Dental Hygiene in the United States (70% superposable curriculum). Differences in the dental degree concepts between China and the United States might lead to inequity situations [16]. Dental education in China is mainly government-supported, with schools providing equipment for students. Some dental schools cannot afford to provide advanced materials for all students [9].

Due to the aforementioned reasons, China exhibits certain differences from other countries, both in the content and discipline orientation of endodontic education. Past research on endodontic teaching primarily focused on undergraduate and postgraduate students, leading to the limitations of research not addressing specialized students and lacking feedback from clinical practitioners. This study is designed as a random sampling survey targeting clinical health care professionals to gather evaluation data on clinical RCT and corresponding teaching practices. This aims to identify issues related to the differences between clinical practices and classroom education, the effectiveness of teaching content, difficulties in practical components, and the bias in educational training.

## Methods

### Questionnaire Design

The design of the questionnaire referred to the fuzzy Delphi method and formed an expert group consisting of experienced oral physician (1), university professor (1), and dental nurses (2). The questionnaire validity was verified through 2 rounds of structured opinion solicitation. Expert discussions focused on the professionalism of the questions (accuracy of instrument references and rationality of operational sequences), representativeness (coverage of essential procedures of standard root canal treatment), and breadth (including preoperative preparation, intraoperative emergencies, and postoperative evaluations). At the same time, the wording of the questions was screened for semantic ambiguity and reading complexity calibration (Flesch-Kincaid level≤8). After 3 iterative modifications, a final consensus rate of 86.4% at the item level was achieved. To prevent respondent misunderstandings, nonconsensual operational terms were annotated in the questionnaire in Multimedia Appendix 1. Participants were required to read the survey instructions before participating in the survey, which outlined the basic process and main content of the questionnaire, as well as clarified the voluntary nature of participation and the noncommercial research purposes of the data.

The questionnaire consists of 30 questions in 4 categories. The basic questions follow a 7-point Likert scale to categorize the sensitivity of the responses. Some questions are designed as multiple-choice and free-response options. The main content focuses on the differences between in-school learning content and clinical practice, the practical difficulty of clinical RCT, and issues related to educational methods and approaches in RCT. The questionnaire refers to the uniformly used endodontic teaching textbooks in the China region, dividing the entire process of clinical root canal treatment into 9 stages: determining indications, x-ray photography and observation, equipment adjustments, local anesthesia, root canal preparation, root canal disinfection and temporary sealing, root canal filling, posttreatment supplies organization, and clinical doctor-patient communication and doctor-nurse cooperation. To prevent any cognitive misinterpretation, we provided annotations for nonconsensus operations (Multimedia Appendix 1). Before participating in the questionnaire survey, participants are required to read the survey notice, which outlines the basic processes and main content of the survey and clarifies the voluntary nature of participation and the noncommercial research purpose of the data.

### Data Source

This study distributed questionnaires through hospital dental specialties and medical forums, covering the Southeastern Region of China.

### Statistical Analysis

Data collection was managed using internet-based survey software. Descriptive statistics were applied to each question to obtain basic distribution characteristics, and data were cross-compared for different grouping situations. The statistical analysis in the study was carried out using SPSS 26 (IBM), with 1-way analysis of variance used for certain differential issues and the K-W independent sample test and nonparametric test methods used for questions related to the difficulty of RCT operations. The level of statistical significance was set at *P*<.05.

### Ethical Considerations

The questionnaire was distributed and collected in May 2024, over a period of 30 days. A total of 90 questionnaires were distributed, yielding 83 valid responses. A total of 7 incomplete or unsuccessfully retrieved questionnaires were excluded from the data analysis, resulting in a response rate of 92.22%. The questionnaire was filled out anonymously, with no personal names or specific hospital names disclosed. This study was conducted in the form of a questionnaire and was approved by the Ethics Committee of Shanghai University of Engineering Science (Approval No. EST-2024‐027). Written informed consent of the participants was obtained prior to enrolment in the study.

## Results

### Overview

**Figure 1.**
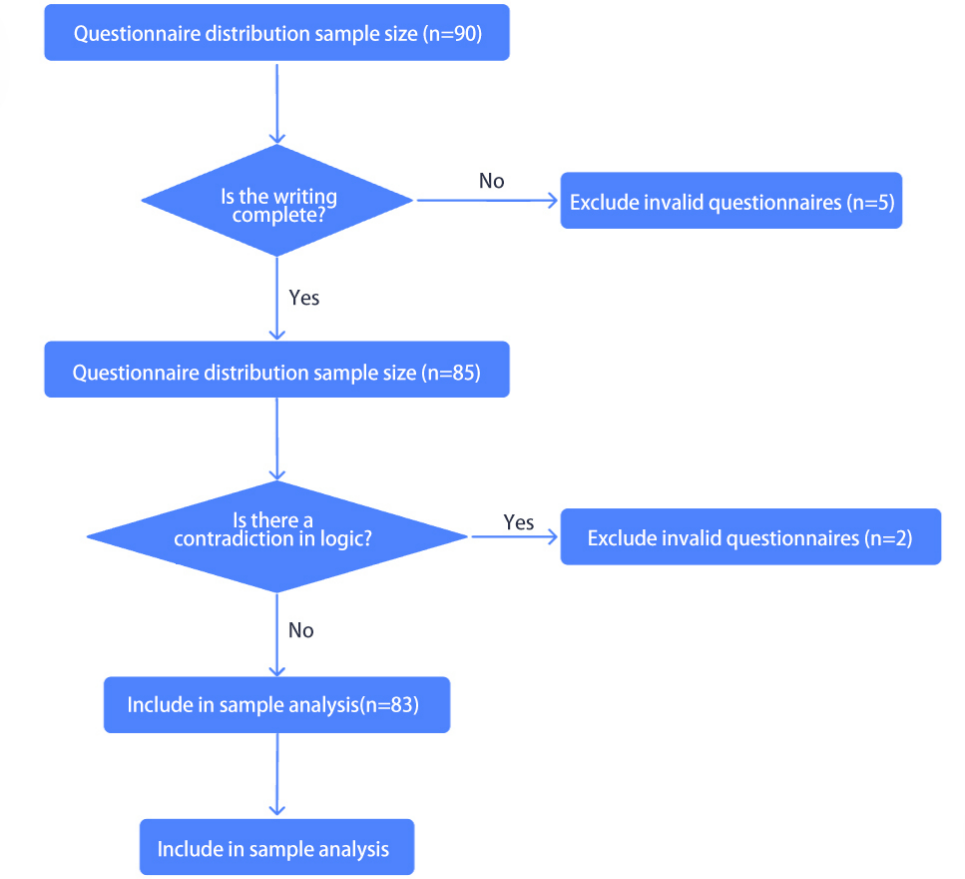
Data screening flowchart.

The questionnaires were distributed and collected from May 1 to May 30, 2024, lasting for 30 days. A total of 90 questionnaires were collected, of which 7 were determined to be invalid due to missing key variables (such as education and age) or logical contradictions. Ultimately, 83 complete data sets were included (missing rate of 7.8%), with an effective recovery rate of 92.22%, as shown in Figure 1. The questionnaires were filled out anonymously without involving personal names or specific hospital names.

Through reliability and validity tests, the standardized reliability coefficient of the questionnaire items was 0.845, and the KMO test coefficient result was 0.87, indicating that the reliability and validity of this questionnaire were good. To evaluate the robustness of the results, the following sensitivity analyses were conducted, using parametric tests (ANOVA) and nonparametric tests (Mann-Whitney U) to analyze the intergroup differences. The results were highly consistent (the direction of *P* values was consistent, and the level of significance did not change). After excluding samples with >10 years of work experience (n=12) and reanalyzing, the impact of education and region on teaching satisfaction remained significant (*P*<.05), indicating that the results were not sensitive to outliers. Therefore, the collected data is meaningful for reference.

**Figure 2.**
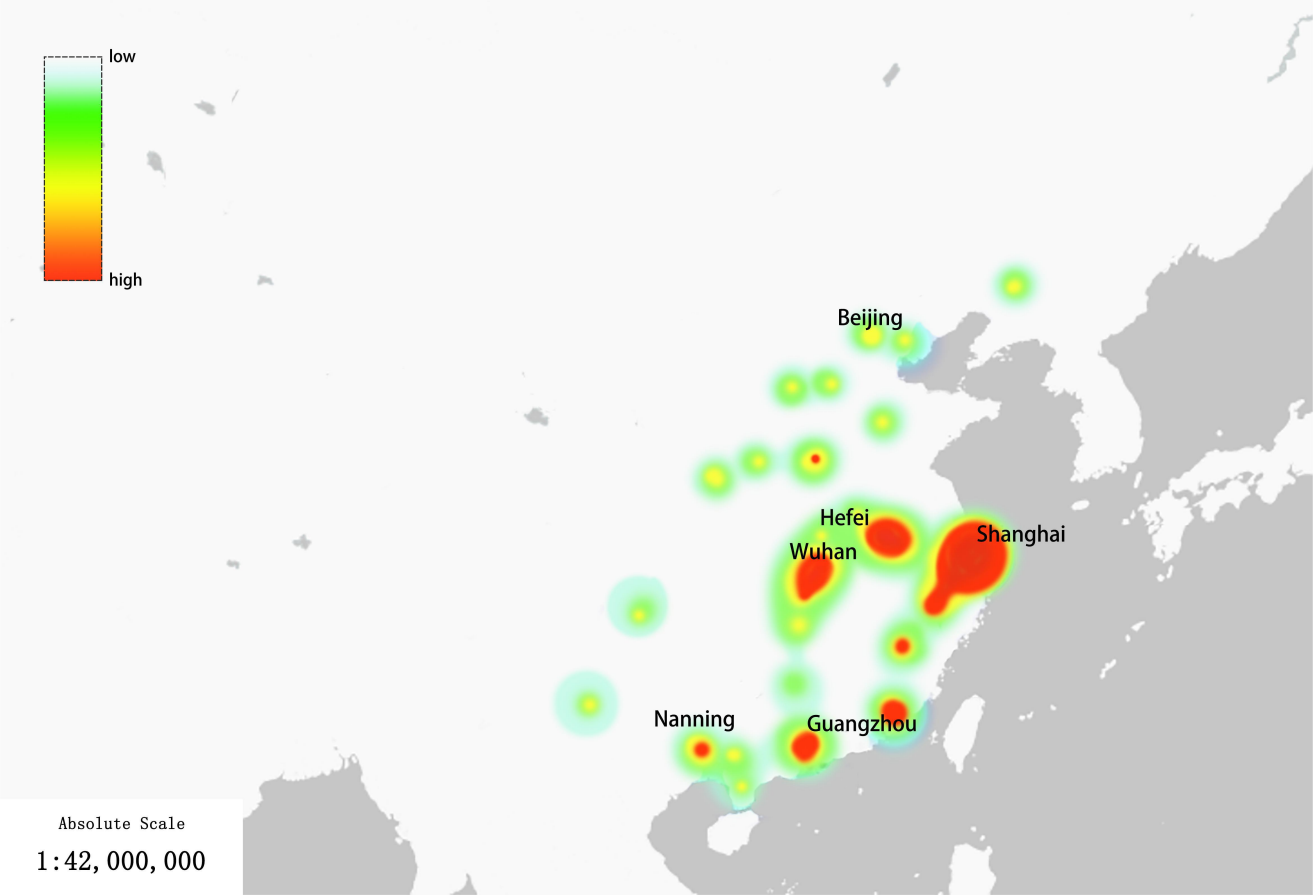
Density visualization of the regional distribution of participants: color indicates the number of participants from less (green) to more (red).

The participants in the study were exclusively practicing dental professionals, with 45.78% hailing from the Shanghai region, as depicted in the density distribution map (Figure 2). These survey participants all have undergone university-level education in the field of dentistry, possess a minimum of 1 year of clinical dental work experience, and have engaged in the performance of RCT during their professional tenure. The age demographic of participants ranged from 21 to 60 years, comprising 36.1% males and 63.9% females. In terms of their educational attainment, 26.5% held junior college degrees, 44.6% possessed bachelor’s degrees, and 28.9% had obtained master’s degrees. The spectrum of clinical dental work experience extended from a minimum of 1 year to a maximum of 36 years.

**Figure 3.**
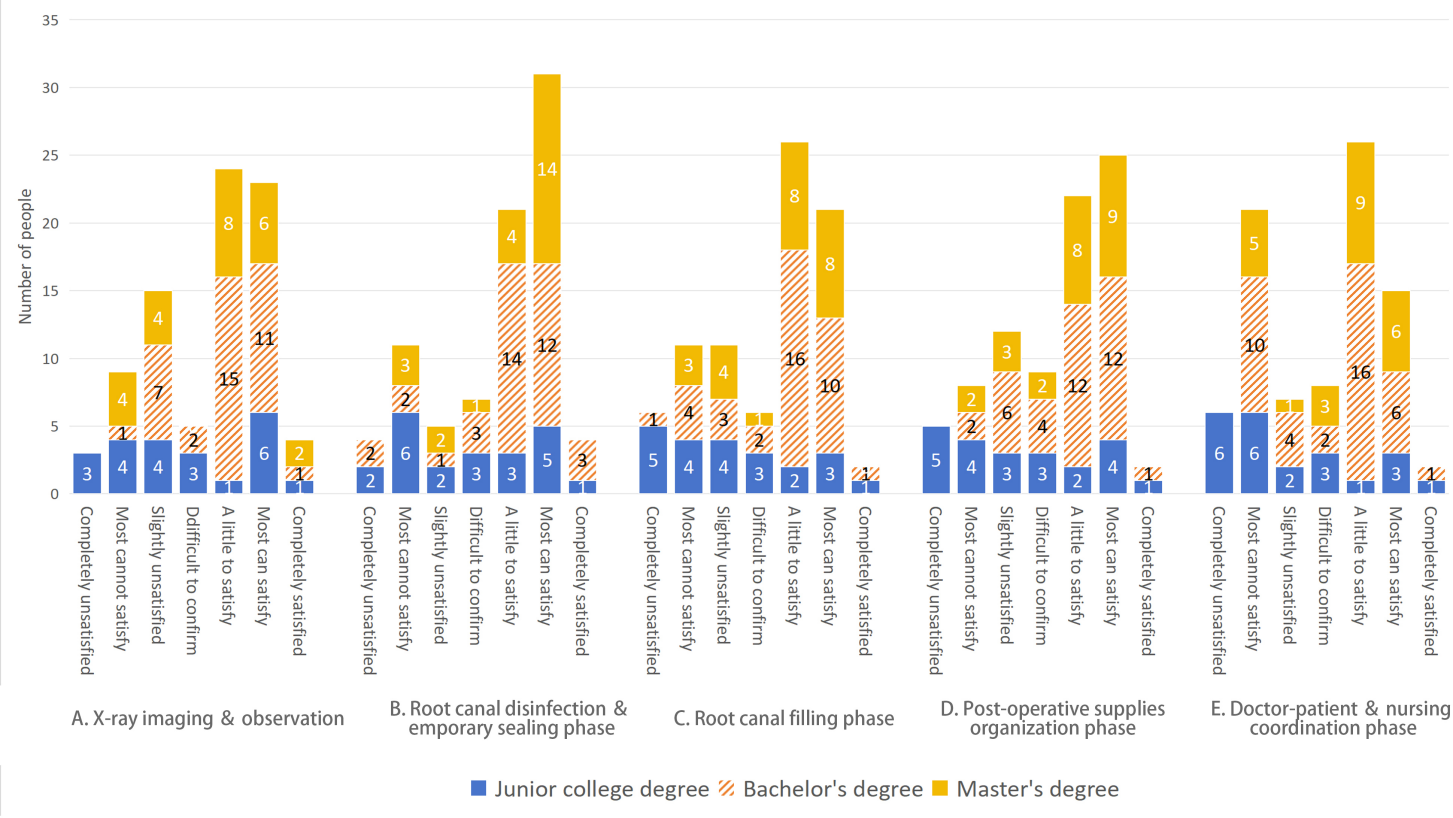
The perceptions of respondents with different educational backgrounds regarding the integration of school teaching and clinical practices across 5 stages of the randomized controlled trial (RCT).

Regarding the congruence between specific aspects of school instruction and clinical practices, respondents’ answers exhibited notable disparities. This investigation revealed that in the x-ray imaging and observation stage, the congruence between bachelor’s and master’s degree holders was notably superior to that of junior college graduates (*P*<.05). Participants with junior college degrees account for 50% (11/22) of the total who expressed negative evaluations of school teaching (selecting “completely unsatisfied,” “most cannot satisfy,” and “slightly unsatisfied” options). In contrast, those with bachelor’s and master’s degrees reported negative evaluations at rates of 22.9% (8/35) and 33.3% (8/24) of their respective totals. Bachelor’s and master’s degree holders significantly surpass junior college degree holders in their positive assessments of school teaching and clinical practices; the proportion of junior college degree holders who gave positive evaluations (choosing “completely satisfied,” “a little to satisfy,” and “slightly satisfied” options) is 36.4% (8/22) of their total, while bachelor’s and master’s degree holders selected positive evaluation options at rates of 77.1% (27/35) and 66.7% (16/24), respectively. Thus, bachelor’s and master’s degree holders evidently hold more favorable views of school teaching and clinical operations compared with those with junior college degrees. For further details, refer to Figure 3. Similarly, discrepancies based on educational backgrounds were also apparent in other phases, such as root canal disinfection and temporary sealing, root canal filling, postoperative supplies organization, doctor-patient and nursing coordination, as depicted in Figure 3.

In Figure 3, junior college degree holders who selected negative evaluation options such as "completely unsatisfied," "most cannot satisfy," and "slightly unsatisfied" constituted 50% (11/22), 45.5% (10/22), 59.1% (13/22), 54.6% (12/22), and 63.6% (14/22) of their demographic, respectively. In contrast, those who opted for positive evaluations like "completely satisfied," "a little to satisfy," and "slightly satisfied" represented 36.4% (8/22), 40.9% (9/22), 27.3% (6/22), 31.8% (7/22), and 22.7% (5/22) of the same group. On the other hand, bachelor’s and master’s degree students who chose negative evaluation options made up 26.2% (16/61), 16.4% (10/61), 24.6% (15/61), 21.3% (13/61), and 32.8% (20/61) of their respective totals, while those who selected positive evaluation options accounted for 70.5% (43/61), 77.0% (47/61), 70.5% (43/61), 68.9% (42/61), and 62.3% (38/61) of their groups*.*

**Figure 4.**
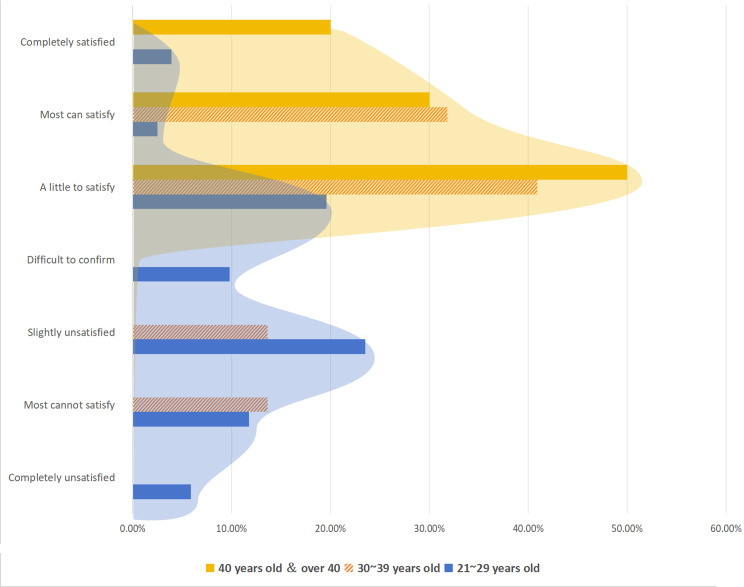
In the context of root canal disinfection and temporary sealing phase, respondents of different age groups expressed their views on the integration of school teaching and clinical operations.

In the phases of root canal disinfection and temporary sealing operations, it is apparent that the “40~” age group perceives a significantly higher alignment between school teaching and clinical practices compared with the “21-29” age group (*P*<.05). Among participants aged 40 and above, 50% (5/10) selected “slightly satisfied,” followed by 30% (3/10) who chose “a little to satisfy” and 20% (2/10) who opted for “completely satisfied.” Conversely, within the 21–29 age group, 23.5% (12/51) of participants indicated “slightly unsatisfied”, which trailed only behind the 25.4% (13/51) who chose the “a little to satisfied” option. For further details, refer to Figure 4. The 40+ age group (yellow) exclusively opted for positive attitudes (choosing “completely satisfied,” “a little to satisfy,” and “slightly satisfied” options), whereas 41.2% (21/51) of the 21‐29 age group (blue) selected negative attitudes (selecting “completely unsatisfied,” “most cannot satisfy,” and “slightly unsatisfied” options).

**Figure 5.**
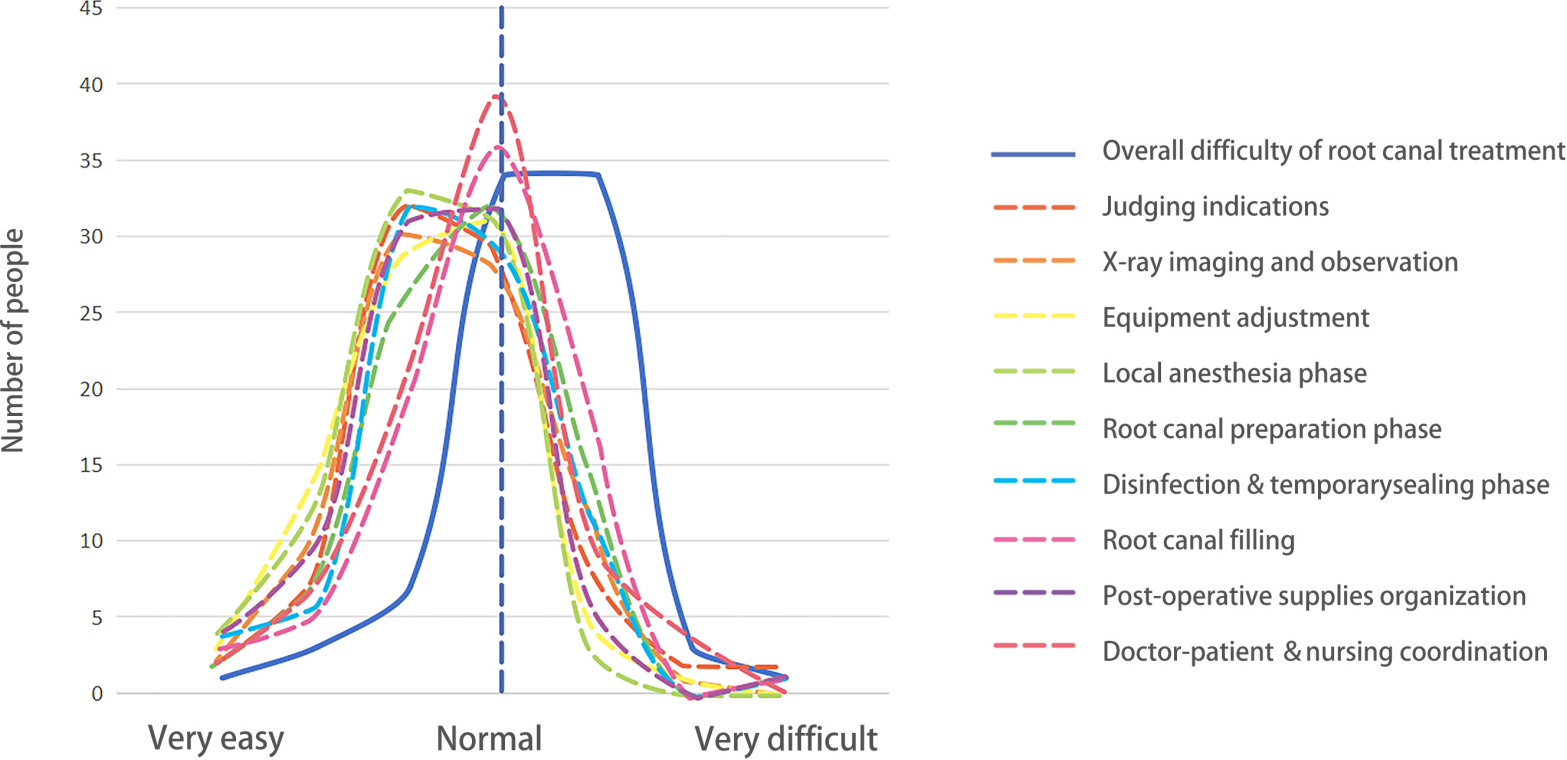
The trend in respondents’ answers to the overall difficulty of root canal treatment and the difficulty of each stage is depicted as follows: the solid line (representing the overall difficulty of a randomized controlled trial [RCT]) has a skewness value of 0.701, which is larger than the skewness values of the dashed lines (representing the difficulty of each individual stage).

On the issue of the practical difficulty of clinical treatment, respondents’ evaluations were homogeneous, revealing no significant categorical disparities. With respect to the overall difficulty of clinical treatment, 82% of respondents (68/83) selected the options of “slightly difficult” and “normal,” exhibiting a skewness value of 0.701. In the assessment of the difficulty of each phase of RCT, respondents generally perceived the difficulty of each stage to lie between “normal” and “slightly easy,” with skewness values lower than the overall difficulty skewness value. Detailed findings are illustrated in Figure 5.

**Figure 6.**
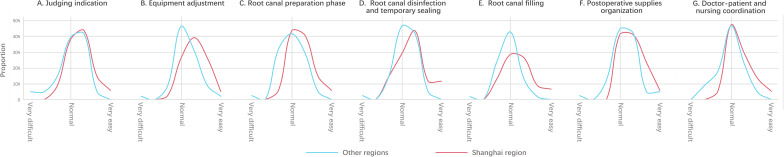
The trend in responses to questions about the variability in certain phases of root canal treatment shows that the skewness values for the Shanghai population samples, from left to right across the 7 graphs (A-G), are 0.385, 0.105, 0.567, 0.323, 0.499, 0.578, and 0.758, respectively. In contrast, the corresponding skewness values for the population samples from other regions are −1.013, −0.339, −0.306, −0.942, −0.383, −0.211, and −0.215.

Regarding perception issues in certain phases, the responses of the participants have exhibited significant variances. For instance, in the equipment adjustment phase, the congruence of the participants from the Shanghai area is generally higher than that of the participants from other regions, and they also perceive the clinical practice difficulty of this phase to be relatively low (*P*<.05). For the composition of the difference in the equipment adjustment phase, refer to Figure 5A: the graph of the sample of the Shanghai area shows a positively skewed distribution, with a skewness value of 0.385; the graph of the sample from other areas exhibits a negatively skewed distribution, with a skewness value of −1.013. In other phases such as indications judgment, equipment adjustment, root canal preparation, root canal disinfection and temporary sealing, root canal filling, postoperative supplies organization, and the phase of doctor-patient and nursing coordination in the clinic, the graphical representation of the sample from the Shanghai area also reveals a significantly positive bias compared with those from other areas, as detailed in Figure 6.

In the survey assessing the disparities between clinical work and on-campus learning, 69.9%(58/83) of the respondents opined that the scenario of judging the indications for RCT in the clinic is more intricate, 68.7% (57/83) of the respondents held the view that there is a marked divergence in the modes and methods of doctor-patient communication in the clinic vis-a-vis the related instruction in the academic setting, and with regard to the configuration and application of equipment, 51.8% (43/83) of the respondents contended that there is a significant variance between the clinical RCT work and the on-campus pedagogy. See for details.

When asked about the teaching methods encountered in the school, 78% (64/83) of the respondents received cavity preparation and filling training in the school, 75.6% (62/83) of the respondents reported that video demonstration was a prevalent teaching method, and 61% (50/83) of the respondents were exposed to the practice of taking x-ray imaging in the school. However, 25.6% (21/83) of the respondents were exposed to dental modeling software practice and digital assessment and review, and only 3.7% (3/83) of the respondents were exposed to VR or AR in the school learning process.

**Figure 7.**
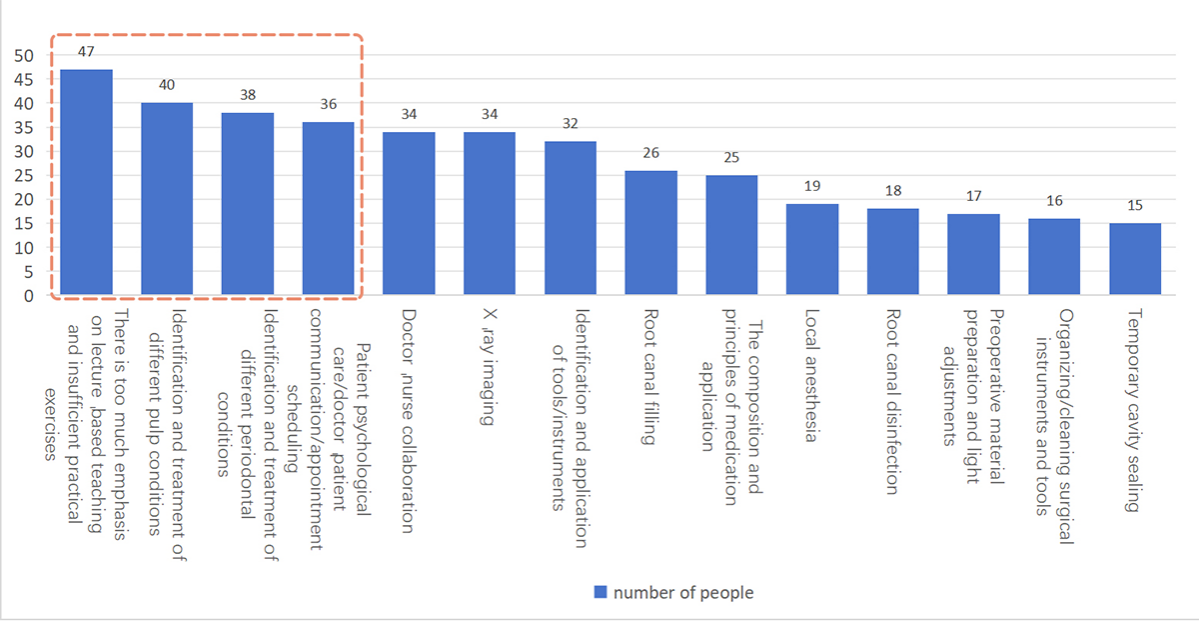
Respondents considered an improved aspect of the school: the red box denotes the 4 aspects of root canal treatment teaching that a significant number of participants suggest should be improved.

56.6% (47/83) of the respondents believed that there is too much didactic teaching in the school teaching process, while the proportion of practical practice is insufficient. 48.2% (40/83) of the respondents advocated for an enhancement in the identification and treatment teaching of different pulp conditions. 45.8% (38/83) of the respondents expressed the desire for an augmentation in the identification and treatment instruction of various periodontal conditions. In addition, respondents who believed that the school should focus on the content of patient psychological care/doctor-patient communication and appointment schedules in the clinic also reached 43.4% (36/83). See Figure 7 for details.

Finally, this study collated and categorized the self-statements of the participants in the “other” option. In the clinical practice of RCT, there is a noted difficulty in identifying or using/disbursing tools or medications: 20% (3/15) of the respondents think it is difficult to use the Gutta Percha Obturation Guns in the root canal filling phase. 33.33% (5/15) of the dental practitioners believed residual pulpitis is a common complication of RCT. In addition, 20% (3/15) of the respondents emphasized that in the on-campus instruction, the importance of the practice instructors cannot be understated, of which 66.67% (2/3) are college degree holders.

## Discussion

### Principal Findings

The results of this study revealed multiple factors affecting the satisfaction of root canal therapy teaching in the Southeastern Region of China, including educational background, regional differences, age stratification, and insufficient periodontal disease teaching content. These findings not only reflect the intuitive feedback of clinical medical staff on teaching effectiveness but also provide important references for optimizing dental education.

### Education Stratification and Uneven Distribution of Teaching Resources

This study shows that medical staff with a specialized degree evaluate the integration of teaching and clinical practice significantly lower than those with undergraduate and master’s degrees. This difference may be closely related to the uneven distribution of teaching resources. In China, most specialized colleges focus their dental courses on basic operation training, while systematic teaching of complex techniques such as root canal therapy is mostly concentrated at the undergraduate level and above. Similar phenomena were also mentioned in the studies of Hua [16] and Jiang et al [17], which pointed out that the number of endodontic class hours in Chinese specialized colleges is only 50%‐60% that of undergraduate colleges, and there is a lack of standardized textbook support. It can be seen that China’s specialized education urgently needs to increase the depth of teaching in root canal therapy to narrow the capability gap between different educational groups.

Further analysis showed that the teaching content of specialized education is insufficient for determining the indications of root canal therapy and dealing with complex root canal anatomical shapes, leading to a lack of systematic thinking when graduates face complex clinical cases [16]. It is worth noting that nearly half of China’s oral medical practitioners have a specialized background [18,19], and their professional abilities directly affect the quality of primary medical care. However, there is very limited research on specialized dental teaching and professional skills. We searched Google Scholar with “pulpology,” “root canal treatment,” “teaching,” and “students” as core keywords. In the related Chinese literature in the past 6 years (2017‐2024), only Hua [16] and Jiang et al [17] mentioned the problem of oral education for people with a specialized degree, while 92.3% (24/26) of the studies focused on people with undergraduate degrees and above. This reflects a serious imbalance between educational research and practical needs.

### Regional Differences Reflect Inequality in Educational Resources

Respondents in the Shanghai area had a significantly higher level of satisfaction with teaching quality compared with other regions, a result highly correlated with regional disparities in the distribution of oral medical resources. A study on national oral health resource surveys also showed issues of inequality in the distribution of the national oral labor force and institutions [19]. Beijing has the highest proportion of dentists, while Tibet has the lowest [15]. In addition to the differences caused by economics between provinces and cities, there are also differences within cities. For example, although the total number of dental care personnel in Shanghai is relatively adequate, its distribution is unfair, with fewer dental care personnel employed in suburban areas [18]. Furthermore, clinical internship bases in the Shanghai area are mostly top-level hospitals, offering students more opportunities to engage with cutting-edge technologies compared with intern hospitals in other regions.

This disparity essentially reflects the synergistic effect of “education-healthcare” resources. In economically developed regions, clinical needs drive the update of teaching content, forming a virtuous cycle. The application of advanced technology in clinical settings leads to the transformation of teaching cases, which in turn enhances student capabilities. While in less developed areas, limited by equipment and teachers, the teaching content lags behind clinical practice [20], resulting in students facing the dilemma of “not taught in school, but needed in clinical practice.”

### Age Factor’s Influence on the Satisfaction of Root Canal Teaching

This study also found significant differences in the perception of certain skills based on age, such as the discrepancy in the root canal disinfection and temporary sealing operation. As shown in Figure 3, the perception of the difficulty of clinical root canal treatment varies with age. The “40 years old and above” group had a significantly higher level of satisfaction with the integration of teaching and clinical practice than the “21–29 years old” group. The “40 years old and above” group generally had a more positive perception of school teaching, while nearly half of the “21–29 years old” group was not very satisfied with school teaching. The reasons for this diversity could be multifaceted. On one hand, over the past 20 years, there may have been changes in the teaching content and methods related to clinical root canal treatment in China’s dental specialty programs. On the other hand, older respondents, due to their longer work experience, may have memory biases about their school teaching and, because of their extensive work experience, may be less sensitive to the perceived difficulty of root canal procedures. In addition, there may be a disconnect between teaching content and clinical practice. Some teaching may not keep pace with clinical changes or emphasize certain operational aspects in school teaching, causing some students to struggle with the clinical practice’s complexity. Further investigation is needed to clarify these situations.

### Interdisciplinary Impact of the Periodontal Disease Teaching Gap

Nearly half of the respondents called for an increase in teaching on differential diagnosis between periodontal and pulp diseases, exposing the current lack of interdisciplinary integration in the curriculum. In other countries, the issue of periodontal disease teaching is also prominent. In the United States and Europe, there is a problem of poor consistency in periodontal disease teaching [21,22]. The conclusions of these previous surveys align with the conclusions of this study, indicating that periodontal disease teaching is a challenge in both oral education and practice. Periodontal disease courses in Chinese schools receive the least amount of time [13]. Due to insufficient class hours, students lack proficiency in key skills related to periodontal diseases, leading to a tendency to overlook periodontal factors in clinical operations and increasing treatment risks.

### New Media Technology

Despite the widely recognized potential of VR or AR technology in dental education [22], only 3.7% of respondents in this study had exposure to such technology. This situation reveals the slow digital transformation in China’s oral education. The reasons for this are two-fold: first, most institutions lack the financial support to purchase VR equipment on a large scale; second, teachers receive inadequate training on the pedagogical suitability of new technologies.

### Other Considerations

In terms of difficulty perception, the results of this study reflected an interesting phenomenon, respondents generally believed that the overall difficulty of root canal treatment was higher than the difficulty of each individual step. As shown in Figure 4, this indicates a lack of “holistic thinking” training in teaching. Currently, most institutions adopt a step-by-step teaching approach (such as practicing root canal preparation and obturation separately), but clinical operations require integrated capabilities across multiple dimensions, such as “case assessment, treatment plan design, instrument selection, and emergency management.” Students lack awareness of the “cascade effects of operational errors” during their school years (such as strategies for managing perforations caused by excessive root canal preparation). Drawing on experiences from other disciplines, Jiangxi University of Traditional Chinese Medicine has adopted “full-process situational simulation” as a teaching method, effectively improving the effectiveness of clinical skills training. It is recommended that China’s dental specialty teaching include “clinical scenario comprehensive training” to train students’ decision-making abilities through high-fidelity models and case libraries, narrowing the gap between “item-by-item proficiency” and “overall competence.”

Due to the wide age range of the samples in this study, there may be differences in teaching methods across different periods. However, after excluding the older sample population, we found that age does not interfere with the impact of other factors (such as education level, region, etc.) on the satisfaction of root canal treatment teaching. Therefore, it suggests that age is not the primary factor, and the disconnection between root canal treatment teaching and clinical practice still exists to some extent.

### Strengths and Limitations

This study adopts the perspective of in-service oral health care workers to retrospectively examine issues in root canal treatment teaching, providing a better understanding of whether school teachings can be effectively applied in clinical practice. The random sampling in this study, without limiting educational background, well reflects the situation and needs of clinical oral health care workers. Moreover, this study uses a Likert 7-point scale, which provides more accurate sensitivity assessments compared with the commonly used 5-point scale.

The main limitation of this study is that the data was primarily collected from the Shanghai area and surrounding cities in the Southeastern part of China, without distinguishing between urban and rural areas, thus preventing further analysis on urban-rural disparities. In addition, due to the limitation of the sample size, the generalizability of the findings from this survey needs to be further improved, necessitating further research to evaluate the effectiveness of school teaching and the details of clinical operations and the difficulty of RCTs.

### Conclusions

This cross-sectional investigation systematically identifies structural discrepancies between preclinical RCT education and clinical workflows in China, with experiential, demographic (eg, training backgrounds), and geographic factors significantly modulating educational-clinical alignment. The findings reveal structural disparities in factors such as clinical experience, educational qualifications, and hospital location. These disparities influence clinical treatment plan decisions, potentially leading to overtreatment or inappropriate management, thus exacerbating patient discomfort, extending treatment duration, and increasing treatment burden. Optimizing classroom content (VR or AR, thermal obturation systems, etc) can expedite students’ transition to clinical practice and bolster their confidence in the diagnostic and therapeutic process. By correlating clinicians’ educational satisfaction metrics with workplace competency demands, this study establishes a novel evaluative framework for dental pedagogy reform, emphasizing competency-based curricula, standardized clinical protocols, and technology-enhanced immersive learning. Subsequent studies can further expand the sample sizes through multicenter sampling across diverse regions to evaluate the changes in academic–clinical consistency.

## Supplementary material

10.2196/65534 Multimedia Appendix 1 Supplementary material.

## Abbreviations

RCT: root canal treatment
