# Supplementary material for: Alignment Between Classroom Education and Clinical Practice of Root Canal Treatment Among Dental Practitioners in China: Cross-Sectional Study
Source: JMIR Med Educ. 2025 Jul 29;11:e65534. doi: 10.2196/65534 (PMC12306950; doi:10.2196/65534)
Supplement: Multimedia Appendix 1 [file mededu-v11-e65534-s001.docx]

Multimedia Appendix 1

**APPENDIX**

**Survey on Root Canal Treatment Learning and Clinical Practice**

**——for Dental Professionals**Please read the Clinical Research Informed Consent Form [Attached] carefully before filling out the questionnaire. Thank you for your participation!

*Basic Information*

1. Please tick your gender

- Male
- Female

1. Please tick your highest level of education ：

- Secondary Vocational
- Junior college Degree
- Bachelor's Degree
- Master's Degree
- Doctoral Degree

1. Please fill in the name of your major studied in school ___________________
2. Year of graduation ____________
3. Years of working in your current job ______ years

(If work experience < 1 year, the questionnaire ends)

1. Have you been clinically involved in root canal treatment related work? (Select "Yes" to continue the questionnaire, select "No" to end the questionnaire)

- Yes
- No

1. Please briefly describe your usual work content:
   You can tick

- Doctor
- Nurse
- Technician
- Guide
- Other, please specify below(You can specify the work content below, e.g., surgical assistance, oral scanning, etc.)

1. How do you perceive the difficulty of performing/assisting in root canal treatment clinically?
   [Please tick the degree you think from the following seven numbers:

| Difficulty Level | Very difficult | Quite difficult | Slightly difficult | Normal | Slightly easy | Quite easy | Very easy |
| --- | --- | --- | --- | --- | --- | --- | --- |
| Number | 1 | 2 | 3 | 4 | 5 | 6 | 7 |

*Questions about the discrepancy between in-school learning content and clinical work*

Do you think that the learning during your time at school meets the content needs of clinical work ?

|  | Very difficult | Quite difficult | Slightly difficult | Normal | Slightly easy | Quite easy | Very easy |
| --- | --- | --- | --- | --- | --- | --- | --- |
| Judging  indications | 1 | 2 | 3 | 4 | 5 | 6 | 7 |
| X-ray imaging  ＆ observation | 1 | 2 | 3 | 4 | 5 | 6 | 7 |
| Equipment  adjustment | 1 | 2 | 3 | 4 | 5 | 6 | 7 |
| Local anesthesia  phase | 1 | 2 | 3 | 4 | 5 | 6 | 7 |
| Root canal  preparation phase | 1 | 2 | 3 | 4 | 5 | 6 | 7 |
| Disinfection ＆  Temporary sealing | 1 | 2 | 3 | 4 | 5 | 6 | 7 |
| Root canal  filing | 1 | 2 | 3 | 4 | 5 | 6 | 7 |
| Post-operative supplies organization | 1 | 2 | 3 | 4 | 5 | 6 | 7 |
| Doctor-patient/nurs-ing coordination | 1 | 2 | 3 | 4 | 5 | 6 | 7 |

*Questions about the practical operation of clinical root canal treatment procedures*

How do you perceive the difficulty of judging the indications for root canal treatment in clinical practice ?

|  | Completely Unsatisfied | Most Cannot Satisfy | Slightly Unsatisfied | Difficult to Confirm | Slightly Satisfied | A Little to Satisfy | Completely Satisfied |
| --- | --- | --- | --- | --- | --- | --- | --- |
| Judging  indications | 1 | 2 | 3 | 4 | 5 | 6 | 7 |
| X-ray imaging  ＆ observation | 1 | 2 | 3 | 4 | 5 | 6 | 7 |
| Equipment  adjustment | 1 | 2 | 3 | 4 | 5 | 6 | 7 |
| Local anesthesia  phase | 1 | 2 | 3 | 4 | 5 | 6 | 7 |
| Root canal  preparation phase | 1 | 2 | 3 | 4 | 5 | 6 | 7 |
| Disinfection ＆  Temporary sealing | 1 | 2 | 3 | 4 | 5 | 6 | 7 |
| Root canal  filing | 1 | 2 | 3 | 4 | 5 | 6 | 7 |
| Post-operative supplies organization | 1 | 2 | 3 | 4 | 5 | 6 | 7 |
| Doctor-patient/nursing coordination | 1 | 2 | 3 | 4 | 5 | 6 | 7 |

*Questions about the methods and approaches of school education in root canal treatment*

1. What do you think is the biggest difference between clinical work and in-school learning in terms of root canal treatment? [Multiple choices]
   A. Drug preparation and identification
   B. Configuration and application of instruments
   C. More complex clinical judgment of root canal treatment indications
   D. Need for coordination with other doctors during the treatment process
   E. More urgent time requirements for clinical operations
   F. Task division in various stages of the surgical process (as an assistant)
   G. Methods of doctor-patient communication
2. Have you been exposed to the following teaching methods in school? If so, please tick. [Multiple choices]
3. Video demonstration
4. 3D printing technology
5. AR/VR technology
6. Modeling software
7. Digital assessment and review
8. Self-assessment/Individualized feedback combined with teacher evaluation
9. Four-handed nursing training
10. Cavity preparation and filling exercises
11. X-ray film shooting
12. Error-based teaching method
13. Which aspects of root canal treatment-related teaching content do you think can be improved in school learning? [Multiple choices]
14. Too much lecture-based teaching, insufficient practical exercises
15. Composition and principles of applied drugs
16. Differentiation and treatment of various periodontal diseases
17. Differentiation and treatment of various pulp diseases
18. Preoperative material preparation and lighting adjustment
19. X-ray film shooting
20. Local anesthesia
21. Identification and application of tools/instruments
22. Root canal disinfection
23. Temporary sealing of the cavity
24. Root canal filling
25. Medical and nursing cooperation
26. Postoperative organization/cleanup of surgical instruments and tools
27. Psychological care for patients/doctor-patient communication/scheduling appointments, etc.
